# RNA viruses in community-acquired childhood pneumonia in semi-urban Nepal; a cross-sectional study

**DOI:** 10.1186/1741-7015-7-35

**Published:** 2009-07-27

**Authors:** Maria Mathisen, Tor A Strand, Biswa N Sharma, Ram K Chandyo, Palle Valentiner-Branth, Sudha Basnet, Ramesh K Adhikari, Dag Hvidsten, Prakash S Shrestha, Halvor Sommerfelt

**Affiliations:** 1 Centre for International Health, University of Bergen, PO Box 7804, N-5020 Bergen, Norway; 2 Medical Microbiology, Department of Laboratory Medicine, Sykehuset Innlandet Lillehammer, Norway; 3 Department of Microbiology, Tribhuvan University Teaching Hospital, Kathmandu, Nepal; 4 Child Health Department, Institute of Medicine, Tribhuvan University, Kathmandu, Nepal; 5 Department of Epidemiology, Division of Epidemiology, Statens Serum Institut, Copenhagen, Denmark; 6 Department of Microbiology and Infection Control, University Hospital of North Norway, Tromsø, Norway; 7 Division of Infectious Disease Control, Norwegian Institute of Public Health, Oslo, Norway

## Abstract

**Background:**

Pneumonia is among the main causes of illness and death in children <5 years of age. There is a need to better describe the epidemiology of viral community-acquired pneumonia (CAP) in developing countries.

**Methods:**

From July 2004 to June 2007, we examined nasopharyngeal aspirates (NPA) from 2,230 cases of pneumonia (World Health Organization criteria) in children 2 to 35 months old recruited in a randomized trial of zinc supplementation at a field clinic in Bhaktapur, Nepal. The specimens were examined for respiratory syncytial virus (RSV), influenza virus type A (InfA) and B (InfB), parainfluenza virus types 1, 2 and 3 (PIV1, PIV2, and PIV3), and human metapneumovirus (hMPV) using a multiplex reverse transcriptase polymerase chain reaction (PCR) assay.

**Results:**

We identified 919 virus isolates in 887 (40.0%) of the 2,219 NPA specimens with a valid PCR result, of which 334 (15.1%) yielded RSV, 164 (7.4%) InfA, 129 (5.8%) PIV3, 98 (4.4%) PIV1, 93 (4.2%) hMPV, 84 (3.8%) InfB, and 17 (0.8%) PIV2. CAP occurred in an epidemic pattern with substantial temporal variation during the three years of study. The largest peaks of pneumonia occurrence coincided with peaks of RSV infection, which occurred in epidemics during the rainy season and in winter. The monthly number of RSV infections was positively correlated with relative humidity (*r*_*s *_= 0.40, *P *= 0.01), but not with temperature or rainfall. An hMPV epidemic occurred during one of the three winter seasons and the monthly number of hMPV cases was also associated with relative humidity (*r*_*s *_= 0.55, *P *= 0.0005).

**Conclusion:**

Respiratory RNA viruses were detected from NPA in 40% of CAP cases in our study. The most commonly isolated viruses were RSV, InfA, and PIV3. RSV infections contributed substantially to the observed CAP epidemics. The occurrence of viral CAP in this community seemed to reflect more or less overlapping micro-epidemics with several respiratory viruses, highlighting the challenges of developing and implementing effective public health control measures.

## Background

Viruses are important causes of lower respiratory tract infection (LRTI) in developing countries [1-3]. The most common cause of viral LRTI is RNA viruses: respiratory syncytial virus (RSV), human parainfluenza virus (PIV), influenza virus, and human metapneumovirus (hMPV). Adenovirus is probably the only DNA virus that is a common cause of LRTI in children [2].

Respiratory viruses, and especially RSV, are leading causes of hospitalization in infants and young children during the cold season in temperate climates [4,5]. Studies in the 1980s highlighted the importance of viruses in LRTI and identified RSV as the predominant cause in children aged <5 years also in developing countries [6]. This was confirmed in a WHO-sponsored denominator-based study of RSV-associated LRTI in four developing countries [7]. Moreover, while influenza virus is being recognized as causing severe LRTI in otherwise healthy children in high-income countries [8], little information on its epidemiology is available from resource-poor settings. A few studies on the more recently discovered hMPV have been published, but these studies were small and covered varying seasons and age groups [9-11]. Seasonality of RSV and influenza in tropical and sub-tropical regions differs from the well-defined seasonal outbreaks seen in temperate climates, and the seasonal pattern of these infections in developing countries varies considerably between regions [1,12]. Knowledge of the local epidemiology of these infections is essential for predicting epidemics and planning preventive measures, such as development and introduction of vaccines in low- and middle-income countries.

Polymerase chain reaction (PCR) is a novel, but now widely applied, method for the detection of respiratory viruses from clinical samples. Compared with conventional methods, PCR has significantly increased sensitivity for respiratory viral diagnosis [13,14] and has also demonstrated high specificity [15,16]. Viral etiology data for community-acquired pneumonia (CAP) from developing countries based on molecular diagnostic methods are scarce, however, and no such studies have to our knowledge been conducted over several years and on a large number of children.

We sought to identify common viral pathogens in CAP in a large number of Nepalese children 2 to 35 months of age visiting a field clinic. We also wished to describe the seasonal pattern of respiratory viral infections over a 3-year period and explore possible associations with available meteorological data.

## Methods

### Study area

Study participants were recruited from the district of Bhaktapur in the Kathmandu Valley, Nepal. A total of 1,913 (86.2%) of the 2,219 cases were recruited from within Bhaktapur municipality, a semi-urban agricultural based town with a population of approximately 80,000, of which we at any time had approximately 4,500 children 2 to 35 months of age under surveillance for respiratory illness. Low income, low dietary intakes and low consumption of dairy and animal products are widespread, as in most parts of Nepal. Malnutrition, mainly manifested as stunting and anemia, are common among children less than 5 years of age [17].

The Kathmandu Valley is situated at an altitude of 1,300 to 1,350 meters above sea level and has a sub-tropical, temperate climate. There are four distinct seasons; pre-monsoon/spring (March to May), monsoon/summer (June to September), post-monsoon/autumn (October to November) and winter (December to February) [18]. Temperatures may rise to 35°C in summer, while minimum temperatures can fall to 0°C in winter.

### Study subjects and case definition

**Figure 1:**
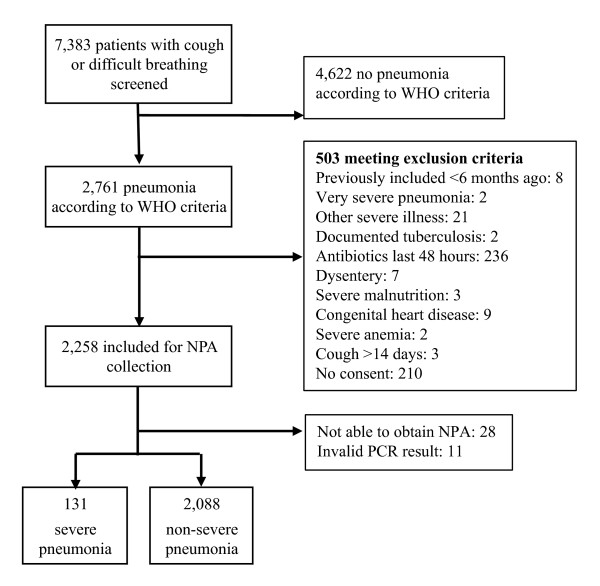
**Study profile for children 2 to 35 months of age included in a study of viral community-acquired pneumonia in Bhaktapur, Nepal, from July 2004 to June 2007**. Severe malnutrition was defined as <70% NCHS (National Center for Health Statistics) median weight for height. Severe anemia was defined as hemoglobin <7 g/dl.

We recruited cases from an open cohort of children less than 3 years of age, who were under monthly active and passive surveillance for respiratory illness. Trained fieldworkers referred children with respiratory complaints to the study clinic at the outpatient department (OPD) at Siddhi Memorial Hospital in Bhaktapur, and families could bring their children for free treatment at our clinic for common childhood illnesses. Children residing in Bhaktapur district, but outside the municipality, were only under such passive surveillance. Children aged 2 to 35 months presenting at our study clinic were screened for fast breathing or lower chest wall indrawing (LCI) and classified according to the standard World Health Organization (WHO) algorithm for acute respiratory infection (ARI) [19] (Figure 1). Pneumonia was defined as cough or difficult breathing combined with fast breathing, that is ≥ 50 breaths/min for children 2 to 11 months old, and ≥ 40 breaths/min for children ≥ 12 months old. Severe pneumonia was defined as cough or difficult breathing combined with LCI. Children with audible or auscultatory wheeze were given 2 doses of 2.5 mg nebulized salbutamol administered 15 min apart followed by reassessment after 30 min, which is in accordance with the revised WHO guidelines [20]. A child was included only if he or she had fast breathing or LCI at reassessment.

During a 3-year period from 29 June 2004 to 30 June 2007, we collected 2,230 nasopharyngeal aspirate (NPA) specimens from equally many cases of pneumonia in 1,909 children (some children were included more than once). These children were, after obtaining informed parental consent, included in a study on zinc as adjuvant therapy for CAP (to be presented elsewhere). All included children were randomized to receive either 10 to 20 mg elemental zinc dispersed in water or placebo tablets daily for 14 days. Cases with very severe pneumonia/disease, that is, cough or difficult breathing with stridor when calm or any general danger signs (inability to drink/breastfeed, persistent vomiting, convulsions, lethargy, or unconsciousness) were not included in the study, but instead referred to a tertiary level hospital after initial treatment. Other exclusion criteria are listed in the study profile (Figure 1). Children could not participate in the study again until after 6 months due to the 6-month follow-up scheme of the clinical trial.

### Field procedures

The child's respiratory rate (RR) was assessed according to WHO guidelines [19], counting twice for 1 min using a UNICEF timer. The lower of the two counts was used in the analyses. Children were weighed using a UNICEF electronic scale (SECA, Hamburg, Germany) accurate to 100 g with a mother/child-function, so the weight of the child could be determined while held by his or her mother. The child's length/height was measured to the nearest 0.1 cm using a wooden measuring board (as recumbent length in children <2 years of age and as height in children ≥ 2 years of age). Oxygen saturation (SpO_2_) was measured either on a finger or a toe with a pulse oxymeter (Siemens MicO2, Siemens Medical Systems Inc, Danvers, MA, USA) using a pediatric sensor (Nellcor, Pleasanton, CA, USA). It was recorded twice 1 min apart after stabilization of the sensor for 1 min. The higher of the two measurements was used in the analyses. The concentration of C-reactive protein (CRP) was determined from a capillary or venous blood specimen using a semi-quantitative rapid test (QuikRead^® ^CRP, Orion Diagnostica, Espoo, Finland) and a portable photometer (QuikRead^® ^101, Orion Diagnostica) according to the manufacturer's instruction. The test had a measurement range of 8 to 160 mg/L and values outside the measurement range were indicated as <8 or >160 mg/L. NPA specimens were obtained using a sterile, disposable suction catheter (Pennine Healthcare Ltd, Derbyshire, UK) with a suction trap (trachea suction set, Unomedical a/s, Birkerød, Denmark) connected to a foot pump (Ambu^® ^Uni-Suction Pump, Ambu A/S, Ballerup, Denmark). The catheter was inserted through the nostril to a distance equivalent to that between the patient's earlobe and nostril [21]. Suction was applied for a minimum of 10 sec with maximum negative pressure of 200 mmHg. Secretion remaining in the catheter after suction was recovered by rinsing 2 to 3 ml virus transport medium (DiagnoStick^®^, Department of Microbiology, University Hospital of North Norway, Tromsø, Norway or UTM, Copan Diagnostics Inc, Corona, CA, USA) through the catheter into the suction trap. The trap was then disconnected and sealed.

### Storage of nasopharyngeal aspirates

The specimens were refrigerated at 2 to 8°C following collection at the field clinic and transported on ice every working day to the main laboratory in Kathmandu, where they were vortexed and divided into three equal aliquots in sterile vials (CryoTubes™, Nunc AS, Roskilde, Denmark). The aliquots analyzed in Nepal were either frozen at -70°C or kept at 2 to 8°C in the refrigerator before analysis. A separate comparative study verified that there were no substantial differences in proportion detected between the two alternative storage temperatures for up to three months (unpublished data). One aliquot was immediately frozen at -70°C and transported to Norway on dry ice and again stored at -70°C in case there should be a need for reanalysis.

### Identification of respiratory RNA viruses

One aliquot of each specimen was tested at our research laboratory in Nepal at the Institute of Medicine, Tribhuvan University, for RSV, InfA and InfB, PIV types 1, 2 and 3, and hMPV using a commercially available multiplex reverse transcriptase PCR assay (Hexaplex Plus^®^, Prodesse Inc, Waukeshaw, WI, USA) with minor modifications of the manufacturer's instructions [22] and according to previous descriptions [15]. In brief, nucleic acids were extracted from 360 μl of NPA (or plasmid RNA from positive control transcripts) using a nucleic acids extraction kit (Roche High Pure Viral Nucleic Acid Kit, F. Hoffman-La Roche Ltd, Basel, Switzerland) according to the manufacturer's instructions. Each run of the assay included a positive RNA control and a negative control (virus transport medium), starting at nucleic acid isolation. Specimens and negative controls were individually spiked with 40 μl of internal control during nucleic acid isolation to identify any inhibitors. cDNA was produced by reverse transcription using random hexamers, murine leukemia virus reverse transcriptase (ABI, Applied Biosystems, Foster City, CA, USA), RNase inhibitor (ABI) and 3 μl of extracted viral RNA. Amplification reactions were performed using GeneAmp^® ^PCR System 2700 (ABI). Ten μl of newly synthesized cDNA was added to a mix consisting of 2.5 U of AmpliTaq^® ^Gold DNA polymerase (ABI) and a Super-Mix containing seven pairs of forward and backward primers flanking unique sequences of the seven viruses (the hemagglutinin neuraminidase gene of PIV types 1, 2 and 3, the matrix protein gene of InfA, the NS1 and NS2 genes of InfB, the NS1 and NS2 genes of RSV and the nucleocapsid gene of hMPV). After initially holding the PCR mixture at 95°C for 10 min, amplification was performed as follows: two cycles at 95°C for 1 min, 55°C for 30 sec and 72°C for 45 sec, and then 38 cycles at 94°C for 1 min, 60°C for 30 sec, 72°C for 30 sec, followed by an additional 7 min at 72°C and immediate cooling to 4°C. After amplification, the PCR products were purified using Qiagen QIAquick PCR Purification Kit (QIAGEN Inc, Valencia, CA, USA) and analyzed by enzyme hybridization assay [15], measuring the optical density at 450 nm (*OD*_450_) using a micro-plate reader (Stat Fax^® ^2100, Awareness Technology Inc, Palm City, FL, USA).

Three hundred and twenty-four NPA aliquots were stored beyond 3 months at 2 to 8°C before analysis in Nepal. Of these, we re-analyzed the 133 that yielded a negative result, now using the aliquot that had been frozen at -70°C and transported on dry ice to Norway. This was done at the Department of Microbiology and Infection Control, University Hospital of North Norway, Tromsø, Norway, using the Hexaplex Plus assay and an automated extraction platform (NucliSens^® ^easyMAG, bioMérieux, Durham, NC, USA). Nucleic acids were extracted from 400 μl of sample, negative and positive processing controls and amplification control using the extraction principle with magnetic particles of this platform.

### Definitions of cut-off values and interpretation of PCR results

The criteria for a positive test were *OD*_450 _≥ 0.400 and at least four times greater than the *OD*_450 _of the negative control [22]. An *OD*_450 _< 0.300 with an *OD*_450 _of the internal control >2.00 indicated a negative test. A reading from 0.300 to 0.399 was interpreted as indeterminate and the sample examined again. If the same result was obtained on repeated testing, the NPA was deemed negative. If the *OD*_450 _of the internal control for a given NPA was <2.00 and sample absorbance was < 0.400 for all tested agents, the NPA was tested again. If the same result was obtained on repeated testing, the interpretation was indeterminate due to potential inhibition, and the case not included in the analyses.

### Data management and statistical analyses

The data were double entered and compared on a daily basis using Microsoft Visual FoxPro version 6.0 (Microsoft Corporation, Redmond, WA, USA). Statistical analyses were performed using Stata/MP 10.0 for Macintosh (Stata Corporation, College Station, TX, USA). The 95% confidence intervals (CI) for proportions were calculated with binominal exact confidence interval using the 'ci' command. Of the children included in this analysis, 274 were enrolled twice and 18 thrice. We used the 'cluster' option in Stata to adjust the confidence intervals of the proportions for repeated enrollments and thus allowed for possible dependence of observations in a child that was included more than once. Anthropometric measures were expressed as Z-scores, which were generated using the WHO Child Growth Standards 2005 [23]. Meteorological data for the Kathmandu airport weather station (located approximately 10 km from Bhaktapur) were obtained from Department of Hydrology and Meteorology, Ministry of Environment, Science and Technology, Kathmandu, Nepal. Mean daily values for relative humidity and temperature were calculated as the average of two daily measurements (relative humidity at 8.45 AM and 5.45 PM, and maximum and minimum temperature). We estimated the Spearman rank order correlation coefficient to describe the association between the monthly number of infections with each virus and meteorological factors.

### Ethical considerations

The study had ethical clearance from the Research Ethics Committee of the Institute of Medicine at Tribhuvan University in Kathmandu and the Regional Committee for Medical and Health Research Ethics of Western Norway. The implementation of the project was in agreement with the international ethical principles for medical research involving human subjects as stated in the latest version of the Helsinki Declaration.

## Results

We excluded 11 cases from the analysis due to inhibition of the PCR. Out of the remaining 2,219 pneumonia cases, 1,263 (56.9%) were boys and 1,016 (45.8%) were infants (<1 year). The mean (SD) age was 13.4 (8.3) months (median 12, inter-quartile range (IQR) 6 to 19) (Table 1). A total of 887 cases (40.0%) tested positive for one or more viruses (CI 37.9%, 42.0%). We identified 919 isolates in NPA from these 887 cases. RSV was the most commonly identified virus with 334 cases out of 887 (37.7%), corresponding to 15.1% of all pneumonia cases (Table 2). Influenza A was detected in 7.4% of all cases and hence was the second most common virus isolated. Severe pneumonia was diagnosed in 131 (5.9%) children and 36 (27.5%) of these had RSV infection. The proportion of samples that was positive for at least one of the seven viruses varied from month to month, ranging from 2.3% to 84.5%.

**Table 1:** Background and clinical characteristics of 2,219 cases of community-acquired pneumonia diagnosed over a 3-year period in Bhaktapur, Nepal

Characteristic	*n*	Value
Demographic		
Age in months	2,219	
Mean (SD)		13.4 (8.3)
2 to 11 months (%)		1,016 (45.8)
12 to 35 months (%)		1,203 (54.2)
Breastfeeding (%)	2,218	1,945 (87.7)
Boys (%)	2,219	1,263 (56.9)
Mean birth weight in grams (SD)^*a*^	1,585	2,856 (464)
Hospital delivery (%)	2,216	1,718 (77.5)
Illiterate (%)^*b*^		
Mother	2,212	578 (26.1)
Father	2,209	116 (5.3)
Father's occupation (%)	2,216	
Agriculture		240 (10.8)
Daily wage earner		1,078 (48.6)
No work		43 (1.9)
Mother's occupation (%)	2,216	
Agriculture		222 (10.0)
Daily wage earner		361 (16.3)
No work outside home		1,472 (66.4)
Symptoms and signs at presentation		
Median number of days with cough at presentation (IQR)	2,219	3 (2 to 4)
Runny nose according to caregiver (%)	1,882	1,632 (86.7)
Mean respiratory rate in breaths/min (SD)		
2 to 11 months	1,016	58 (5.4)
12 to 35 months	1,203	49 (6.5)
Axillary temperature (%)	2,218	
≥ 37.5°C		908 (40.9)
≥ 38.5°C		293 (13.2)
Wheezing (%)^*c*^	2,219	994 (44.8)
Crepitations (%)	2,219	651 (29.3)
Lower chest indrawing (%)	2,219	131 (5.9)
Oxygen saturation (%)	2,219	
<93%		670 (30.2)
<90%		42 (1.9)
Median CRP in mg/L (IQR)	2,215	15 (<8, 28)
Mean hemoglobin in g/dl (SD)	2,218	11.1 (1.2)
Anthropometric		
Mean weight for length Z-score (SD)	2,212	-0.26 (1.0)
Mean length for age Z-score (SD)		-1.1 (1.2)
Length for age <-2Z-scores (stunted) (%)^*d*^		526 (23.8)
Weight for length <-2Z-scores (wasted) (%)^*d*^		82 (3.7)

^*a*^Written documentation from 1,273 birth certificates, remaining 312 from mother's recall;^*b*^defined as no schooling/not able to read and write;^*c*^wheezing prior to administration of nebulized salbutamol; ^*d*^the WHO Child Growth Standards 2006 [23].

CRP = C-reactive protein; IQR = inter-quartile range; SD = standard deviation.

**Table 2:** Distribution of the different RNA viruses in 2,219 cases of community-acquired pneumonia in children 2 to 35 months of age diagnosed at a field clinic in Bhaktapur, Nepal, from July 2004 to June 2007

	Number of isolates	All pneumonia cases	Virus positive cases
		(*n *= 2,219)	(*n *= 887)
Virus	*n*	% (95% CI)	% (95% CI)
RSV	334	15.1 (13.6 to 16.6)	37.7 (34.5 to 40.9)
Influenza A	164	7.4 (6.3 to 8.6)	18.5 (16.0 to 21.2)
PIV type 3	129	5.8 (4.9 to 6.9)	14.5 (12.3 to 17.0)
PIV type 1	98	4.4 (3.6 to 5.4)	11.0 (9.1 to 13.3)
hMPV	93	4.2 (3.4 to 5.1)	10.5 (8.5 to 12.7)
Influenza B	84	3.8 (3.0 to 4.7)	9.5 (7.6 to 11.6)
PIV type 2	17	0.8 (0.4 to 1.2)	1.9 (1.1 to 3.1)

CI = confidence interval; hMPV = human metapneumovirus; PIV = parainfluenza virus; RSV = respiratory syncytial virus.

Infection with more than one virus was identified in 29 (3.3%) of the 887 positive specimens. The most common double infection was PIV3 in combination with hMPV, which was found in nine cases. We identified three viruses in one case (PIV1, PIV2, RSV) and four viruses in another (PIV1, PIV2, InfB, RSV).

**Figure 2:**
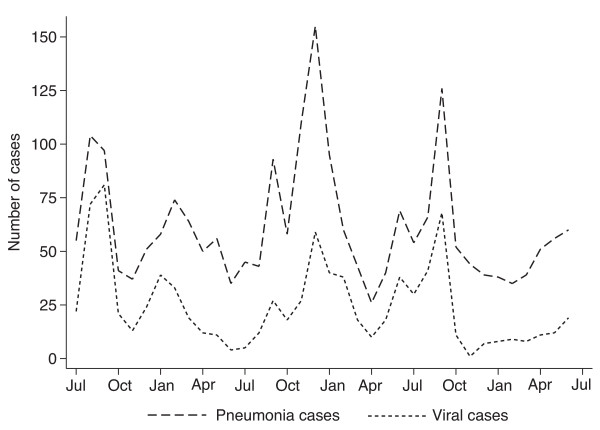
**Monthly number of community-acquired pneumonia cases and cases with a positive virus PCR in children aged 2 to 35 months identified at a field clinic in Bhaktapur, Nepal, from July 2004 to June 2007**.

**Figure 3:**
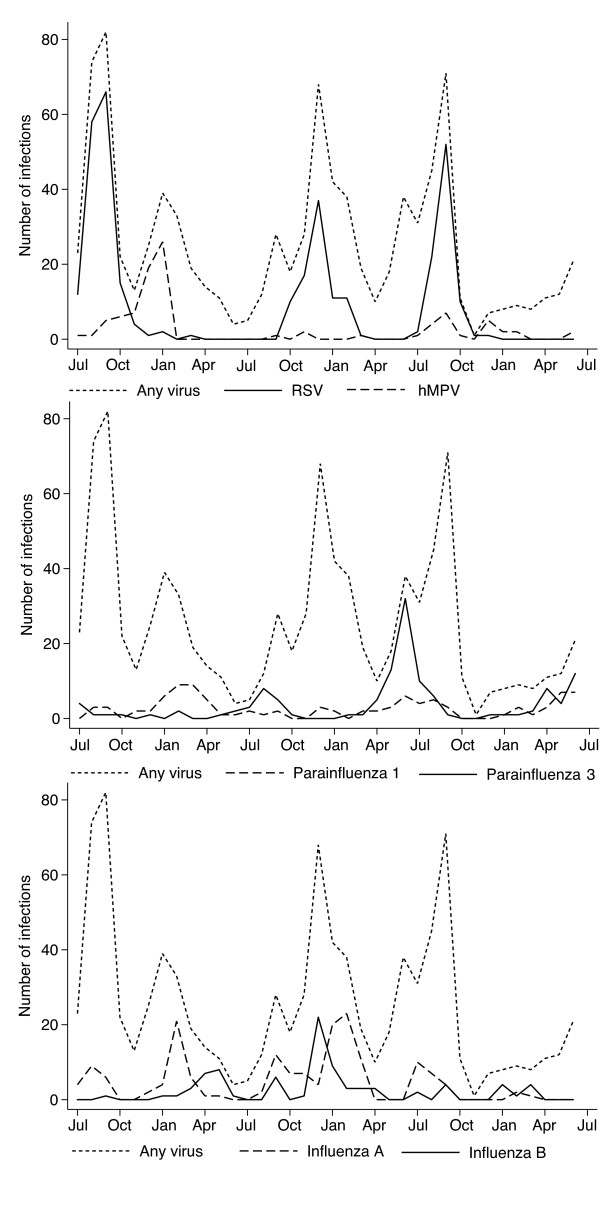
**Monthly number of viral isolates in nasopharyngeal specimens from 2,219 cases with community-acquired pneumonia in children 2 to 35 months of age identified at a field clinic in Bhaktapur, Nepal, from July 2004 to June 2007**. Parainfluenza 2 isolates were not included in the graph due to few positive cases.

The occurrence of CAP during the course of our study exhibited substantial temporal variation and a clear epidemic pattern (Figure 2). In each of the three years, we observed a sharp increase in the occurrence of pneumonia at the end of the monsoon season in August to September. There were also significant CAP epidemics during winter, the first year peaking in February and the second year in December, but we observed no winter epidemic in the third year. There were also smaller peaks of CAP in spring. Epidemics of infection with individual respiratory viruses contributed to literally all of these CAP epidemics, such as the three RSV epidemics (the second of which was compounded by epidemics with InfA and InfB infections), an InfA epidemic superimposed on an hMPV epidemic, and a PIV3 epidemic (Figure 3). Displays of the spatial-temporal distribution of these individual epidemics are visualized in [24]. PIV1 infections occurred in small numbers throughout the year, whereas only 17 cases of PIV2 infection were seen during the entire study period.

**Figure 4:**
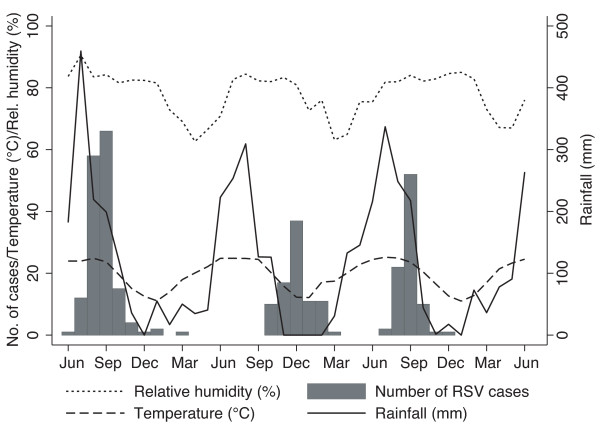
**Monthly number of RSV infections in children aged 2 to 35 months with community-acquired pneumonia from 29 June 2004 to 30 June 2007, in Bhaktapur, Nepal, depicted with monthly variation of relative humidity, rainfall and temperature**. The mean daily measurement for the month was used for relative humidity and temperature, while rainfall was calculated as the total measurement for the month.

The monthly distribution of RSV infections and meteorological data are shown in Figure 4. The monthly number of RSV infections was positively correlated with relative humidity in the Spearman's correlation analysis, but not with temperature or rainfall (Table 3). The same association with relative humidity was seen for hMPV. In contrast, the number of PIV3 was positively associated with both temperature and rainfall, and not with relative humidity. InfA did not correlate with any of these meteorological factors, while InfB showed moderate negative correlation with all three factors.

**Table 3:** Spearman's correlation coefficients for association between monthly number of different viral infections and meteorological factors

Virus	Relative humidity (%)^*a*^	Temperature (°C)^*a*^	Rainfall (mm)^*b*^
RSV	0.40 (*P *= 0.015)	-0.058 (*P *= 0.74)	-0.061 (*P *= 0.72)
Influenza A	0.023 (*P *= 0.89)	-0.034 (*P *= 0.084)	-0.12 (*P *= 0.47)
PIV type 3	-0.088 (*P *= 0.61)	0.65 (*P <*0.0001)	0.68 (*P <*0.0001)
PIV type 1	-0.38 (*P *= 0.024)	0.17 (*P *= 0.31)	0.23 (*P *= 0.18)
Influenza B	-0.34 (*P *= 0.045)	-0.31 (*P *= 0.066)	-0.39 (*P *= 0.019)
hMPV	0.55 (*P *= 0.0005)	-0.15 (*P *= 0.37)	0.022 (*P *= 0.90)
PIV type 2	0.15 (*P *= 0.39)	0.39 (*P *= 0.019)	0.30 (*P *= 0.078)

^*a*^Mean daily measurement for the month; ^*b*^total measurement for the month.

hMPV = human metapneumovirus; PIV = parainfluenza virus; RSV = respiratory syncytial virus.

## Discussion

This is to our knowledge the largest epidemiological study in almost four decades of childhood CAP in a developing country that identifies several common viruses. We isolated at least one viral pathogen using PCR from 40% of children during the 3-year study period. The literature reveals few studies based on sensitive molecular diagnosis from similar resource-poor settings. Apart from recent studies on hMPV, most previous studies in developing countries have not used PCR for virus detection [1].

Hospital-based studies in children <5 years of age from developing countries published over the last 20 years have identified viruses (excluding measles) in 8.4% [25] to 45% [26] of LRTI episodes. One of the larger studies, which included nearly 1,500 Pakistani children <5 years of age, detected a virus (including adenovirus) in 37% of cases using viral culture and immunofluorescence (IF) [27]. Previous community-based studies with longitudinal follow-up of children have identified viruses in 11% to 45% of LRTI cases [1,28,29].

Not unexpectedly, RSV was by far the most common virus in our study, identified in 15.1% of all pneumonia cases and 37.7% of viral positive cases. Etiology studies in developing countries that included viruses have identified RSV in a median of 20% (5th to 95th percentile 1 to 53) of LRTI cases [30] and in 6% to 96% (mean 39%) of LRTI cases with a viral etiology [1]. It should be noted that many of these studies also included measles. A recently published denominator-based study from a birth cohort comprising 635 Kenyan children followed through three RSV epidemics reported that 13% of cases with LRTI were attributable to RSV infection, which was diagnosed using IF [31]. In India, RSV accounted for 17% of hospitalized cases with LRTI in New Delhi [32] and 7% of LRTI cases included in a 3-year longitudinal community study in Haryana [28]. After RSV, InfA and PIV3 were the most common viruses in our study, as reported elsewhere [1,3,28]. We detected hMPV in 4.2% of pneumonia cases. It is estimated that MPV worldwide accounts for 5 to 7% of ARI in young children requiring hospitalization [33]. In India, hMPV was detected in 3.2% of 662 children hospitalized for ARI over a 2-year period from April 2005 [34].

RSV and influenza virus are known to occur in well-defined recurrent epidemics during the cold season in temperate climates [1,2]. In tropical and subtropical areas, RSV infections have been reported to peak more often in relation to the wet season, but locations close to the equator show a less consistent pattern, some with almost continuous RSV activity and varying seasonal peaks [1,7]. Studies in the Indian subcontinent have reported RSV infections to peak both in the cold season [27] and in the rainy season [35,36], as well as being detectable throughout the year [35,37]. We observed RSV epidemics both in winter and during the monsoon. The virus was isolated during a period of 6 to 7 months and the epidemic peaks were observed with intervals of 15 and 9 months, which is consistent with patterns described elsewhere [31,38].

We detected influenza in 25 of the 36 study months. The largest influenza peaks occurred in winter from December to February in the first two years, but we also isolated influenza during the monsoon period. A summer outbreak of influenza has previously been reported from Nepal [39]. Surveillance data from 2007 at the Kathmandu sentinel site has showed that InfB was prevalent throughout the year except in May, and that InfA prevailed during June to August and reappeared in December [40]. Unlike in temperate regions, where influenza occurs in well-defined outbreaks lasting 2 to 3 months once a year in the winter, influenza is detectable for a greater part of the year in tropical and sub-tropical regions and the timing of outbreaks is less predictable [41,42]. Influenza peaks have been reported to occur during periods of high rainfall in many tropical locations [12].

We observed one distinct hMPV epidemic, which peaked between December 2004 and January 2005. The epidemic lasted from July 2004 to January 2005 and comprised 70% of the hMPV isolates detected during the entire 3-year study period. Substantial variation in the yearly incidence of hMPV has also been reported earlier [43,44]. Epidemics with this virus have been reported to occur during late winter to spring in temperate climates [33,45], including North India [46], where the majority of hMPV were detected from December to February, and Korea [45], where hMPV peaked between February and April. A study in subtropical Hong Kong found hMPV infections mainly in the spring and summer months [47], similar to that of RSV [48] and occasionally influenza [49].

We isolated PIV3 in 27 months of our 36 month-study, but an increasing number of infections were seen between April to May and September each year, while the largest epidemic occurred in June 2006. The virus has been known to exhibit an endemic pattern and to cause yearly epidemics in spring and summer in temperate climates [50]. PIV1 was isolated in smaller numbers throughout the study period (in 28 of 36 months), and we could not observe the biennial pattern of fall epidemics seen elsewhere [51].

As could be observed from the displays of the occurrence of viral CAPs in the study children [24], the spatial-temporal distribution of these infections revealed more or less overlapping micro-epidemics with several respiratory viral pathogens spreading between households in Bhaktapur.

Despite two of the three RSV epidemics occurring during the summer monsoon, neither rainfall nor temperature was significantly associated with RSV infections in our study, while there was a positive correlation with relative humidity. In tropical Asian countries, somewhat contradictory patterns of associations between RSV and meteorological factors have been observed. In Hong Kong, the monthly incidence of RSV infection was positively correlated with both temperature and relative humidity [48], whereas in Malaysia, the number of RSV infections was inversely correlated with temperature [52]. RSV infections peaked towards the end of the rainy season during the first and third year of our study. We explored whether there could be an association between preceding rainfall and RSV infections, and by introducing a 2-month lag after peak precipitation, such an association could indeed be identified (data not shown).

A strong association with relative humidity was also found for hMPV infections. Association with meteorological factors has not previously been described for this virus. Belonging to the *Pneumovirinae *subfamily [53], both RSV and hMPV could require similar conditions for transmission and infectivity. In contrast, PIV3 was strongly and positively correlated with both temperature and rainfall, but not with relative humidity. A study in Singapore found no such associations for PIV3 [54]. It is also interesting to note that, in our study, InfB infections showed an inverse correlation with relative humidity, temperature and rainfall, while InfA infections did not correlate with any of these meteorological factors. In Singapore, InfA outbreaks were also not associated with meteorological factors, but InfB infections were reported to positively correlate with rainfall [54].

As derived from the correlation analyses (*r*_*s *_= 0.68), up to 46% of the monthly variation in occurrence of viral infections in our study could be explained by meteorological factors. However, the underlying reasons for the observed seasonal patterns demonstrated by these individual respiratory viruses are unclear. Climate could have a direct impact on virus survival, transmission efficiency, and host immunity, or have an indirect effect through climate-dependent behavior change, such as indoor crowding and eating habits [55]. It is likely that several factors interact in complex ways in the development of observed epidemics under favorable climatic conditions and that the contribution of individual factors varies for the different viruses.

We detected more than one virus in 3.3% of the virus-positive NPA specimens, which is similar to what is reported using molecular methods detecting the same viruses as in our study [56], but lower compared with studies using molecular methods detecting a wider array of viruses [57,58]. The frequency of viral co-detections varies widely and depends on the number of diagnostic methods applied [59] and the number of pathogens tested for [58]. By employing multiplex PCR assays for 11 viruses, a study in 515 Korean children aged ≤ 5 years detected viruses in 312 (60.6%) of cases and a viral co-detection in 36 (11.5%) [45]. In addition to the seven viruses identified in our study, the Korean study also detected adenovirus, coronavirus, rhinovirus, and bocavirus; and, notably, the last virus was identified in 22 of the 36 co-detections. The clinical role of bocavirus in pneumonia in otherwise healthy children is unclear [60,61], as is the importance of multiple viral infections [57,59]. Quantitative PCR methods to determine viral load in clinical specimens could provide valuable information on the pathogenetic role of each virus in such co-detections [61], as could case-control studies.

Compared with pneumonia, pulmonary tuberculosis is not common in young Nepalese children [62]. To avoid including cases of undiagnosed tuberculosis and reactive airway disease, we did not include children that had been coughing for more than 14 days and we reassessed respiratory rate in wheezers after salbutamol administration before diagnosis of pneumonia [20]. The number of measles cases in Nepal has decreased dramatically since 2003 [63]. Measles vaccination is recommended at 9 months and the vaccine coverage among those that were 9 months or older in our study area was >90% (unpublished data). Moreover, less than 1% of our cases had a rash and all of these were above 1 year of age. Thus, we believe that our sample consisted of children with pneumonia, and not tuberculosis, bronchial asthma, or measles.

Despite the high sensitivity and specificity of the Hexaplex Plus assay [15], we may have underestimated the number of viral infections. Viral load in a specimen depends on several conditions, such as time of collection in relation to onset of illness. In most of the study children, the NPA specimen was collected early in the course of illness, which increases the likelihood of detecting an infectious agent [14]. Moreover, by not including children who had taken antibiotics during the last 48 hours, we primarily recruited new cases of pneumonia. Using multiplex instead of single molecular assays may have contributed to an underestimation of the number of viral CAP cases, as some loss of sensitivity is an inherent limitation of multiplex PCR assays [13].

We observed a large month-to-month variation in the proportion of CAP cases from whom we identified a respiratory RNA virus, largely reflecting the epidemic pattern of viral infections in this sub-tropical setting. A limitation of our study was that we were not able to include more etiologic agents in our diagnostic panel. Adenovirus is among the more common viral respiratory pathogens causing LRTI in preschool children [64]. Atypical bacteria, like *Mycoplasma pneumoniae*, cause pneumonia more frequently in school-age children, but can also cause mild infections in younger children [65]. *Streptococcus pneumoniae *and *Haemophilus influenza *are the main bacterial pathogens causing childhood pneumonia. However, nasopharyngeal carriage of these bacteria is frequent in healthy, young children [66]. Moreover, their detection in pneumonia is hampered by the difficulties of obtaining specimens from the lower airways and the low sensitivity of identifying these pathogens by blood culture [67], and such invasive methods are not well suited for community settings. *S. pneumoniae *has been suggested to play a role in the development of virus-associated pneumonia in children in hospital, especially in cases with influenza and PIV types 1 to 3 [68]. The monthly number of cases where we did not identify any virus was particularly pronounced in the CAP epidemic that peaked in December 2005 (Figure 2), which comprised the largest outbreaks of influenza A and B (Figure 3). There is abundant evidence that infection with influenza viruses predisposes for pneumococcal disease [69]. Additionally, we cannot rule out the possibility that some of the cases where we detected a viral pathogen in fact had a mixed viral-bacterial pneumonia or pneumonia after acquisition of a new pneumococcal serotype during an upper respiratory viral infection. Again, quantitative PCR methods and carefully conducted case-control studies could shed light on the clinical importance of detecting viral pathogens in NPA specimens from children with pneumonia.

## Conclusion

We detected a viral pathogen from the nasopharynx in 40% of the children with CAP in this study, indicating that respiratory RNA viruses play an important role in this common childhood illness in Nepal. RSV was the most common agent isolated, and occurred with considerable temporal variation. As for many other common childhood infections, such as diarrhea, the occurrence of viral CAP in this community seemed to reflect more or less overlapping micro-epidemics with several respiratory viruses, highlighting the challenges of developing and implementing effective public health control measures in resource-poor settings.

## Abbreviations

ARI: acute respiratory infection; CAP: community-acquired pneumonia; CI: confidence interval; CRP: C-reactive protein; hMPV: human metapneumovirus; IF: immunofluorescence; InfA: influenza virus type A; InfB: influenza virus type B; IQR: inter-quartile range; LCI: lower chest wall indrawing; LRTI: lower respiratory tract infection; NPA: nasopharyngeal aspirate; OPD: outpatient department; PIV1: parainfluenza virus type 1; PIV2: parainfluenza virus type 2; PIV3: parainfluenza virus type 3; RR: respiratory rate; RSV: respiratory syncytial virus; SpO_2_: oxygen saturation.

## Competing interests

The authors declare that they have no competing interests.

## Authors' contributions

MM participated in the protocol design, planning, field implementation, microbiological analyses, data management and analysis, and wrote the first draft of the manuscript. TAS was the overall coordinator of the project, participated in the protocol design, funding, planning, data management, analysis and preparation of the manuscript. BNS participated in the planning and implementation of microbiological analyses and data management. RKC participated in the protocol design, planning, field implementation, data management, analysis and preparation of the manuscript. PVB participated in the protocol design, planning, field implementation, data management, analysis and preparation of the manuscript. SB participated in the protocol design, planning, field implementation, data management, analysis and preparation of the manuscript. RKA participated in the protocol design, planning and preparations of the manuscript. DH participated in the planning and implementation of the microbiological analyses, interpretation of data and preparation of the manuscript. PSS participated in the protocol design, planning and field implementation. HS participated in the protocol design, planning, analysis and preparation of the manuscript. All authors approved the final version of the manuscript.

## Pre-publication history

The pre-publication history for this paper can be accessed here:


